# Olfactory dysfunction and amyloid-positivity in Parkinson’s disease—longitudinal analysis of cognitive decline and cerebrospinal fluid markers

**DOI:** 10.1371/journal.pone.0325560

**Published:** 2025-08-21

**Authors:** Victoria Larsson, Sara Hall, Kaj Blennow, Oskar Hansson

**Affiliations:** 1 Clinical Memory Research Unit, Department of Clinical Sciences Malmö, Lund University, Lund, Sweden; 2 Skåne University Hospital, Department of Neurology and Rehabilitation Medicine, Lund, Sweden; 3 Memory Clinic, Skåne University Hospital, Malmö, Sweden; 4 Department of Psychiatry and Neurochemistry, Institute of Neuroscience and Physiology, The Sahlgrenska Academy at the University of Gothenburg, Gothenburg, Sweden; 5 Clinical Neurochemistry Laboratory, Sahlgrenska University Hospital, Mölndal, Sweden; 6 Paris Brain Institute, ICM, Pitié-Salpêtrière Hospital, Sorbonne University, Paris, France; 7 Neurodegenerative Disorder Research Center, Division of Life Sciences and Medicine, and Department of Neurology, Institute on Aging and Brain Disorders, University of Science and Technology of China and First Affiliated Hospital of USTC, Hefei, P.R. China; National Center of Neurology and Psychiatry (NCNP), JAPAN

## Abstract

**Background:**

Olfactory dysfunction is a common non-motor symptom in Parkinson’s disease (PD). The objective was to evaluate the association between olfaction in PD with cross-sectional and longitudinal assessments of clinical variables and novel cerebrospinal fluid (CSF) markers.

**Methods:**

Patients with PD and baseline olfactory function assessed using the Brief Smell Identification Test (B-SIT) were included from the BioFINDER-1 cohort. Clinical variables, CSF measures and disease status were assessed longitudinally for up to 11 years. CSF was analyzed using Roche Elecsys® NeuroToolKit, including biomarkers of neurodegeneration, glial activation, neuroinflammation and the core Alzheimer disease biomarkers.

**Results:**

A total of 172 patients with PD were included, 63 with normal olfactory function and 109 with hyposmia. No differences were seen in clinical variables at baseline. Glial fibrillary acidic protein was the only CSF marker differing at baseline, being elevated in hyposmic patients with PD (12.25 ± 3.87 vs 10.46 ± 3.68, p = 0.001). At follow-up, olfactory function declined predominantly in patients with normal olfaction at baseline (β = −0.25 [−0.40 to −0.12], p = 0.001). Patients with PD with both olfactory dysfunction and amyloid-positivity (defined by the CSF Aβ42/Aβ40 ratio) declined faster in several cognitive and motor measures. Olfaction and amyloid-status were independently associated with increased risk of progressing to dementia (B-SIT score, HR = 0.77 [0.67–0.88] and amyloid-positivity, HR = 4.47 [2.30–8.67]).

**Conclusions:**

Olfactory dysfunction and amyloid-positivity are independently associated with a higher rate of cognitive decline and progression to dementia in patients with PD. Novel CSF markers of neurodegeneration and glial-activity do not differ depending on olfactory status in PD.

## Introduction

Parkinson’s disease (PD) is the second most common neurodegenerative disease affecting 2–3% of the population over the age of 65 [1], with increasing incidence globally in the last decade [2]. In addition to the characteristic motor symptoms in PD, several non-motor features are recognized [3]. Olfactory dysfunction is one of the most common non-motor symptom with a reported prevalence ranging from 50% to 90% [4], sometimes predating motor complaints by several years [5]. The early manifestation is thought to represent α-synuclein deposits in the olfactory bulb as one of the induction sites for disease, which is then followed by spreading through the brainstem and the cerebral cortex [6,7]. Further, hyposmia has in a neuropathological study been shown to distinguish PDD, including PDD versus Alzheimer’s disease (AD) with high accuracy [8].

This high prevalence and ease of testing have generated interest in using olfaction as a biomarker in PD. However, most studies have been cross-sectional, relatively small in size and have demonstrated equivocal findings [9]. Olfaction does seem to decrease over time, particularly in early disease, but there are degrees of fluctuation and an unclear relationship with disease duration [10]. Whilst some studies demonstrate that olfactory function in PD is associated with motor [11], psychiatric [12] and cognitive measures [11,13–15], others have not seen any clear correlations [9,10,16]. Still, a few studies have implied that hyposmia in PD is associated with an increased risk of progression to both mild cognitive impairment (MCI) [15] and dementia [17,18].

Only two studies have examined the potential association between cerebrospinal fluid (CSF) markers and olfactory function in PD, again with contradictory findings. The studies have assessed core AD CSF biomarkers, with one study finding lower CSF amyloid-beta 42 (Aβ_42_) levels in patients with PD and worse olfaction, with no changes in total tau (T-tau) or phosphorylated tau (P-tau) [15]. Findings in another study were discrepant, with higher CSF Aβ_42_, lower T-tau but no difference in P-tau in patients with PD with olfactory dysfunction compared to those with normal olfaction [19]. The latter study also analyzed total α-synuclein, demonstrating higher levels in CSF in patients with PD and olfactory dysfunction, whilst other studies have showed decreasing α-synuclein levels in the CSF in PD compared to healthy controls [20].

In addition to the core CSF AD-biomarkers, some of the most promising novel CSF biomarkers are related to neurodegeneration and microglial or astroglial activation, specifically soluble triggering receptor expressed on myeloid cells 2 (sTREM2), glial fibrillary acidic protein and YKL-40 (also known as chitinase 3-protein 1) as well as S100 calcium-binding protein B (S100b). This is appealing given the growing body of evidence suggesting that neuroinflammation, particularly that mediated by microglia, may be a feature of the pathogenesis in PD [21,22]. The Elecsys® NeuroToolKit (NTK, Roche Diagnostics International Ltd, Rotkreuz, Switzerland) is a fully automated immunoassay CSF biomarker panel offering measures of neurodegeneration, glial activity, neuroinflammation as well as core AD-biomarkers, used to demonstrate alterations in AD cohorts [23–26], with less evidence in patients with PD [27,28].

We aimed to further assess the relationship of olfactory dysfunction in PD using longitudinal data from a prospective cohort with up to 11 years follow-up. More specifically, we aimed to assess the relationship between olfactory function, clinical variables (motor, cognitive, autonomic, psychiatric) and novel CSF markers, as well as the impact on disease progression to dementia.

## Methods

### Participants

Participants were included from the Swedish BioFINDER-1 cohort (NCT01208675, for details see biofinder.se) at Lund University. For this study, participants had to have a diagnosis of PD and have measurements of olfactory function at baseline. All participants fulfilled NINDS criteria for possible or probable Parkinson’s disease at baseline [29]. Exclusion criteria were the following i) age above 85 years, ii) presence of generalized malignancy, iii) ongoing or earlier advanced abuse of alcohol or illicit drugs, iv) presence of clinically diagnosed Alzheimer’s dementia, vascular dementia or frontotemporal lobe dementia, v) presence of severe psychiatric disorders, vi) presence of other severe neurological disease, vii) participation in a clinical drug trial within the last 30 days. All participants underwent a medical history, complete neurologic examination and neuropsychological testing. Participants were enrolled between October 2008 to June 2016. All participants gave written informed consent, and ethical approval was granted by the Regional Ethical Committee in Lund, Sweden, (dnr 290/2008). The study was conducted according to the Helsinki Declaration.

### Clinical measures and progression to dementia

Participants were followed longitudinally with yearly follow-ups for up to 4 years, and then biannually for up to 10 years, including cognitive testing and detailed physician assessments. The Brief Smell Identification Test (B-SIT®; Sensonics, Inc., Haddon Heights, NJ) [30] is a widely used and validated 12-item non-invasive test of olfactory function. The total B-SIT scores range between 0–12 where higher scores represent better olfactory function, with abnormal olfaction being classified as B-SIT scores ≤ 8 [30,31].

Cognitive assessments included the mini-mental state examination (MMSE) as measure of global cognitive function [32], ten-word delayed recall test from the Alzheimer’s Disease Assessment Scale-Cognitive Subscale (ADAS DW recall) [33] measuring episodic memory and A Quick Test (AQT) [34] assessing attention and processing speed. Motor function was evaluated using Unified Parkinson’s Disease Rating Scale Part III (UPDRS-III) [35] and Hoehn and Yahr (H&Y) scale [36]. Scales for Outcomes in Parkinson’s Disease – Autonomic Dysfunction (SCOPA-AUT) [37] is a patient-reported questionnaire assessing autonomic symptoms in PD. Patients were classified according to different PD phenotypes; postural instability and gait difficulty (PIGD) or tremor-dominant (TD) [38]. The hospital anxiety and depression scale (HADS) was used for psychiatric symptoms [39].

Diagnosis at follow-up was based on study visit assessments, the treating physician’s assessment and medical chart review by physicians experienced in movement disorders and cognitive medicine. The diagnosis of dementia was based on the DSM-5 criteria for major neurocognitive disorder. None of the participants fulfilled criteria for dementia at baseline.

### CSF measurements

CSF was collected and handled according to a structured protocol as previously described [40]. All CSF biomarkers (T-tau, phosphorylated tau at threonine-181 [P-tau], Aβ_40_, Aβ_42_, neurofilament light [NfL], neurogranin, α-synuclein, soluble triggering receptor expressed on myeloid cells 2 [sTREM2], glial fibrillary acidic protein [GFAP], YKL-40 [also known as chitinase 3-protein 1], S100 calcium-binding protein B [S100b], interleukin 6 [IL-6]) were measured using robust prototype assays as part of the Roche Elecsys® NeuroToolKit on cobas e411 and e601 instruments (Roche Diagnostics International Ltd, Rotkreuz, Switzerland). All measurements were performed at the Clinical Neurochemistry Laboratory, University of Gothenburg, Sweden by board-certified laboratory technicians who were blinded to diagnostic and other clinical data. For this study, all participants were classified as either amyloid β-peptide (Aβ) positive or negative according to a predefined cut-off of CSF Aβ_42_/Aβ_40_ ratio below 0.066. The methods of α-Syn RT-QuIC analyses have been described in detail elsewhere [41].

### Statistical analyses

Baseline group comparisons were performed with age-adjusted analysis of variance for continuous variables and chi square for categorical variables. CSF values were winsorized at the 99^th^ percentile to avoid extreme outliers and group comparisons were performed using Mann-Whitney U test. Multiple linear regression models were applied to assess the relationship between baseline continuous clinical variables. Longitudinal trajectories of individual variables were assessed using linear mixed effects (LME) models, using random slopes and intercepts, with baseline age, sex and group (normal or hyposmia) as an interaction with time as covariates (R-package lme4). The assumption of normality was satisfied.

Cox proportional hazard regressions were applied to assess risk of progression to dementia. Univariate analyses were performed, followed by fast backward variable selection (R-package rms). This method uses the fitted complete model and computes approximate Wald statistics by computing conditional (restricted) maximum likelihood estimates assuming multivariate normality of estimates, allowing model reduction and subsequent multivariable analyses. The proportional hazards assumption was satisfied.

All analyses were performed with the statistics software R (version 4.2.0, R Core Team 2022]. All CSF values were log-transformed and scaled in regression models. FDR correction was applied to the p-values using the Benjamin-Hochberg procedure. Statistical significance was set at p < 0.05.

Pseudonymized data will be shared by request from a qualified academic investigator for the sole purpose of replicating procedures and results presented in the article and if data transfer agrees with EU legislation on the general data protection regulation and decisions by the Swedish Ethical Review Authority and Region Skåne, which should be regulated in a material transfer agreement. Data requests can be made through bf_executive@med.lu.se.

## Results

### Participants and baseline demographics

A total of 172 patients with PD were included in this study, 63 with normal olfactory function (B-SIT > 8) and 109 with hyposmia (B-SIT ≤ 8.). Baseline demographics are outlined in Table 1. Age-adjusted group comparisons were applied given that patients with hyposmia were older than those with normal olfactory function. No differences were seen in the clinical variables between the two groups at baseline. Multiple linear regression models showed no association between baseline B-SIT scores and disease duration, baseline cognitive scores, motor scores, depression, anxiety or autonomic symptoms.

**Table 1:** Baseline demographics.

Variable	PD normal olfactory function (n = 63)	PD hyposmia (n = 109)	p-value
Age, baseline (sd)	62.22 (11.96)	66.94 (8.35)	0.003
Sex, female (%)	28 (44)	37 (109)	ns
Education, years (sd)	13.13 (3.59)	12.67 (4.21)	ns
*APOE-ε4*, carrier (%)	20 (33)	32 (30)	ns
-missing	2	1	
Disease duration, years (sd)	3.17 (4.40)	3.30 (4.72)	ns
Disease type			
- PIGD, n (%)	20 (32)	51 (47)	ns
- TD, n (%)	36 (57)	50 (47)	
- Unclear, n (%)	7 (11)	6 (6)	
- missing	0	2	
Smoker, yes (%)	2 (3)	5 (5)	ns
Symptoms of cold at baseline			
- No, n (%)	58 (92)	97 (90)	ns
- Mild, n (%)	3 (5)	5 (5)	
- Upper airways infection, n (%)	2 (3)	5 (5)	
- missing	0	2	
B-SIT, score (sd)	9.94 (0.88)	5.64 (1.77)	<0.0001
Orthostatic hypotension, yes (%)	12 (19)	27 (25)	ns
-missing	1	2	
Hypertension, yes (%)	18 (29)	23 (21)	ns
-missing	1	1	
Hyperlipidemia, yes (%)	3 (5)	5 (5)	ns
Diabetes, yes (%)	3 (5)	2 (2)	ns
-missing	0	1	
SCOPA-AUT, score (sd)	12.01 (7.99)	13.20 (7.82)	ns
HADS anxiety, sub score (sd)	4.38 (4.18)	5.13 (4.13)	ns
HADS depression, sub score (sd)	3.33 (3.25)	4.14 (3.37)	ns
UPDRS-III, score (sd)	16.11 (10.33)	17.30 (10.48)	ns
Hoehn & Yahr, score (sd)	1.75 (0.83)	1.98 (0.80)	ns
MMSE, score (sd)	28.30 (1.30)	28.00 (1.89)	ns
ADAS DW recall, score (sd)	2.63 (1.81)	3.38 (2.24)	ns
AQT color form (sd)	66.57 (15.79)	72.11 (19.96)	ns

Age-adjusted group comparisons were applied. Abbreviations: ADAS DW recall, Alzheimer’s Disease Assessment Scale-Cognitive Subscale delayed word recall. AQT, A Quick Test. B-SIT, brief smell identification test. HADS, hospital anxiety and depression scale. MMSE, mini-mental state examination. PIGD, postural instability and gait difficulty. PD, Parkinson’s disease. SCOPA-AUT, Scales for Outcomes in Parkinson’s disease – Autonomic Dysfunction. TD, tremor-dominant. UPDRS-III, Unified Parkinson’s Disease Rating Scale Part III.

The only CSF biomarker differing at baseline was GFAP, found to be elevated in patients with PD with hyposmia compared to those with normal olfactory function (12.25 ± 3.87 vs 10.46 ± 3.68, p = 0.001, Table 2, Fig S1, Supporting material). Multiple linear regression models showed no significant associations between baseline B-SIT scores and baseline CSF biomarkers.

**Table 2:** CSF markers at baseline.

Variable	PD normal olfactory function (n = 63)	PD hyposmia (n = 109)	p-value
αSyn RT-QuIC positive (%)	38 (72)	90 (93)	0.001
-missing	10	12	
Amyloid positive*, yes (%)	8 (15)	21 (22)	ns
-missing	9	12	
CSF α-synuclein, pg/mL (sd)	162.69 (76.70)	164.21 (63.52)	ns
CSF Aβ_40,_ ng/mL (sd)	15.45 (4.26)	15.03 (4.31)	ns
CSF Aβ_42,_ pg/mL (sd)	1316.25 (476.19)	1274.77 (537.72)	ns
CSF GFAP, ng/mL (sd)	10.46 (3.67)	12.25 (3.87)	0.001
CSF IL-6, pg/mL (sd)	4.23 (1.41)	4.73 (1.80)	ns
CSF Neurogranin, pg/mL (sd)	583.74 (201.30)	592.51 (238.86)	ns
CSF NfL, pg/mL (sd)	119.32 (54.96)	142.86 (103.03)	ns
CSF P-tau, pg/mL (sd)	15.80 (15.96)	15.96 (4.99)	ns
CSF S100b, ng/mL (sd)	1.22 (0.26)	1.20 (0.27)	ns
CSF sTREM2, ng/mL (sd)	9.96 (2.64)	10.44 (2.80)	ns
CSF T-tau, pg/mL (sd)	184.28 (52.84)	189.92 (57.82)	ns
CSF YKL-40, ng/mL (sd)	159.30 (64.00)	164.95 (54.55)	ns

Age-adjusted group comparisons were applied. *CSF Aβ_42_/Aβ_42_ ratio below 0.066. Abbreviations: Aβ, beta amyloid. αSyn RT-QuIC, alpha-synuclein real-time quaking induced conversion. GFAP, glial fibrillary acid protein. IL-6, interleukin 6. NfL, neurofilament light. PD, Parkinson’s disease. P-tau, phosphorylated tau at threonine-181. S100b, S100 calcium-binding protein B. sTREM2, soluble triggering receptor expressed on myeloid cells 2. T-tau, total tau. YKL-40, chitinase 3-protein 1.

### Longitudinal changes in olfactory function

**Fig 1:**
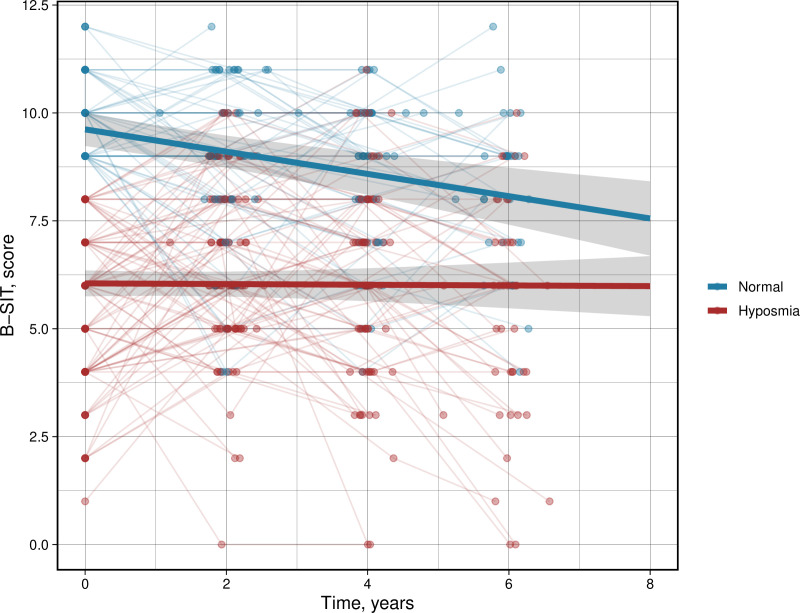
Longitudinal trajectories of B-SIT over time in participants with Parkinson’s disease and either normal olfactory function or hyposmia at baseline. Bold lines represent the predicted trajectory in each group.

The median follow-up of cognitive variables in the cohort was 5.76 years (SD 2.78) with no significant difference between patients with PD with normal olfactory function and hyposmia. There was no overall decrease in B-SIT scores in the entire group over time (β = −0.11, CI −0.18 to −0.04, p = 0.004), with a more pronounced effect observed in those individuals with normal olfactory function at baseline compared to those with hyposmia (β = −0.25, CI −0.40 to −0.12, p = 0.001, Fig 1).

### Olfactory dysfunction and longitudinal changes in clinical variables

LME models were constructed to assess the effect of olfactory function at baseline and the association with longitudinal change in clinical variables. Although a more rapid decline of MMSE was seen in participants with hyposmia (β −0.32, 95% CI −0.54 to −0.10, p = 0.005), this finding was not significant after FDR correction. No other significant associations were seen between baseline olfactory function and longitudinal trajectories of ADAS delayed word recall, AQT, UPDRS, H&Y, SCOPA-AUT, HADS, or any of the CSF biomarkers (Fig S2 in Supporting material).

**Fig 2:**
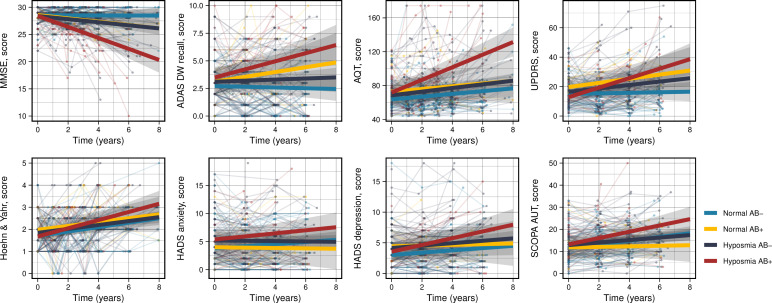
Longitudinal trajectories of cognitive, motor, autonomic and psychiatric scores over time in participants with Parkinson’s disease depending on olfactory function and amyloid status. Bold lines represent the predicted trajectory in each group. MMSE, mini-mental state examination. ADAS DW recall, Alzheimer’s Disease Assessment Scale-Cognitive Subscale delayed word recall. AQT, A Quick Test. HADS, hospital anxiety and depression scale. SCOPA-AUT, Scales for Outcomes in Parkinson’s disease – Autonomic Dysfunction. UPDRS-III, Unified Parkinson’s Disease Rating Scale Part III. AB + , amyloid-positive. AB-, amyloid-negative.

Subgroup analyses were performed depending on amyloid status. This demonstrated that participants with hyposmia who were also amyloid-positive had worsening in MMSE (β −1.01, 95% CI −1.37 to −0.65, p < 0.0001), ADAS DW recall (β 0.40, 95% CI 0.12–0.68, p < 0.01), AQT color form (β 5.82, 95% CI 3.64–8.00, p < 0.0001) and UPDRS-III (β 3.02, 95% CI 1.39–4.66, p < 0.001) over the follow-up time (Fig 2). This contrasted with participants who were amyloid-negative with hyposmia or had normal olfactory function at baseline irrespective of amyloid status. There were no significant associations between either group and longitudinal changes in H&Y, SCOPA-AUT, HADS or any of the CSF biomarkers.

### Olfactory dysfunction is associated with progression to dementia

**Fig 3:**
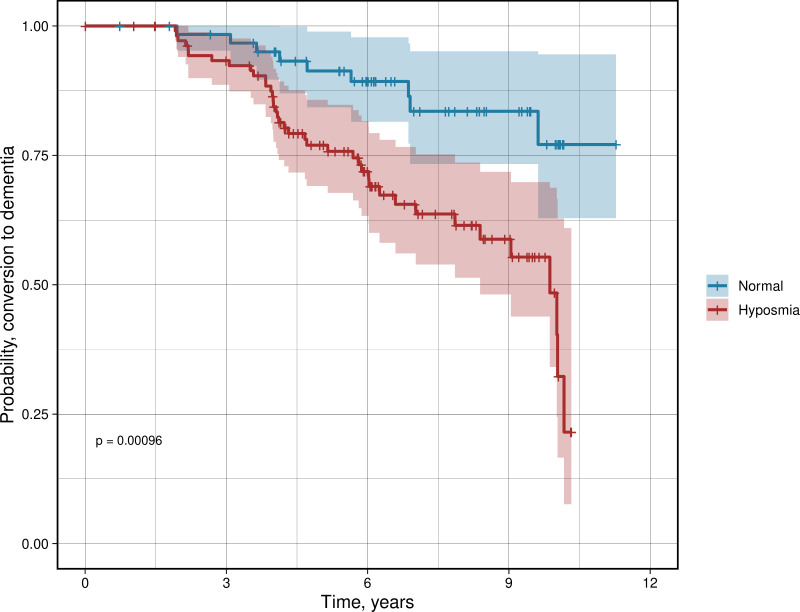
Progression to dementia. Kaplan-Meier curve demonstrating probability of converting to dementia in Parkinson’s disease depending on olfactory function at baseline.

The mean clinical follow-up time was 6.25 years, with participants who had normal olfactory function being followed for slightly longer than those with hyposmia (6.88 ± SD 2.55 years versus 5.89 ± SD 2.48 years, p = 0.05). Participants with hyposmia at baseline had a higher rate of conversion to dementia during follow-up compared to normal olfactory function (Fig 3), with an associated hazard ratio (HR) of 2.47 (95% CI 1.17–5.20) when corrected for age and sex. In the group with hyposmia a total of 39 (36%) converted to dementia, compared to 9 (14%) in the group without hyposmia. Converters to dementia had a clinical phenotype compatible to PDD, however one patients was assessed as PDD with vascular changes and one as PD with vascular dementia at clinical follow up.

Out of the other available variables, age- and sex-adjusted Cox proportional hazard models showed significant HR only for age at baseline (HR 1.09, 95% CI 1.05–1.13, p < 0.0001), amyloid-positivity (HR 3.94, 95% CI 2.06–7.52, p < 0.0001), B-SIT score (HR 0.83, 95% CI 0.74–0.93, p = 0.002), SCOPA-AUT (HR 1.06, 95% CI 1.02–1.10, p = 0.004) and AQT color form (HR 1.02, 95% CI 1.00–1.04, p = 0.004). Associations were also seen with HADS anxiety, ADAS DW recall, *APOEe4* and CSF NfL, however these were not significant after FDR correction, see Table S1 in Supporting material.

Backwards step-down model selection was performed to identify the most important variables relevant for dementia conversion. This was performed first for clinical variables, where the final factors in the model were B-SIT score (HR 0.77, 95% CI 0.67–0.88, p < 0.001) and amyloid-positivity (HR 4.47, 95% CI 2.30–8.67, p < 0.0001) when adjusted for age and sex (Table 3). The second model selection included all CSF markers, where only CSF Aβ_42_/Aβ_40_ (HR 0.63, 95% CI 0.48–0.83, p < 0.001) was deemed important for the final model (Table 3).

**Table 3:** Multivariate Cox proportional regression hazard modelling.

Model	Variables	Beta	SE	HR (95% CI)	p-value
1. Clinical^†^	Age baseline	0.07	0.02	1.07 (1.03-1.12)	<0.01
	Sex	−0.37	0.33	0.69 (0.36-1.32)	ns
	B-SIT, score	−0.26	0.07	0.77 (0.67-0.88)	<0.001
	Amyloid positivity*	1.50	0.34	4.47 (2.30-8.67)	<0.0001
2. CSF^‡^	Age baseline	0.08	0.02	1.08 (1.04-1.13)	<0.001
	Sex	−0.53	0.33	0.59 (0.31-1.14)	ns
	Aβ42/Aβ40	−0.46	0.14	0.63 (0.48-0.83)	<0.001

*CSF Aβ42/Aβ42 ratio below 0.066. B-SIT, brief smell identification test. ^†^Deleted variables education, APOE4 carrier, disease duration, smoking status, orthostatic hypotension, MMSE, ADAS DW recall, AQT, HADS anxiety, HADS depression, SCOPA-AUT, UPDRS-III, Hoehn &Yahr. ^‡^Deleted variables α-syn, GFAP, neurogranin, IL-6, NfL, Aβ42, Aβ40, P-tau, S100, sTREM2, T-tau, YKL40. Abbreviations: Aβ, beta amyloid. B-SIT, Brief smell identification test. MMSE, mini-mental state examination.

## Discussion

In the present study, we examined olfactory dysfunction in a longitudinal prospective cohort of patients with PD, specifically looking at clinical variables, CSF markers and progression to dementia. The main results show that the combination of olfactory dysfunction and amyloid-positivity (low CSF Aβ_42_/Aβ_40_ ratio) is associated with an increased risk of decline in cognitive and motor function. Specifically, patients with PD and both olfactory dysfunction and amyloid-positivity at baseline had a decline in global cognition (assessed by MMSE), memory function (ADAS DW recall), processing speed (AQT color form) as well as motor function (UPDRS-III) over the follow-up time. Olfactory dysfunction and markers of amyloid-pathology were associated with an increased risk of progression to dementia. Other clinical variables such as autonomic or psychiatric measures, as well as CSF biomarkers reflecting neurodegeneration, neuroinflammation or glial activity, did not significantly impact the analyses. Contrary to previous studies [42], we did not see that cognitive measures at baseline alone contributed to lower performance in olfactory function tests in PD. Other studies have also been able to demonstrate how cognition impacts the within-subject irreproducibility on specific smell test items [43], something which could however add value for future studies.

Finding hyposmia to be a relevant marker of progression to dementia was expected, given that this has been seen in several previous studies [15,17,18]. Adjusting the analyses also for markers of concomitant amyloid-pathology, another recognized predictor for progression to dementia in PD [44–48], demonstrated an independent risk of progression to dementia with worse olfactory function at baseline. This is in line with previous results showing that a composite measure of olfaction and CSF Aβ_42_ was a significant predictor of conversion to MCI [15]. This result is of importance in clinical settings, where the identification of patients with both features may indicate worse prognosis. Notably, no other CSF markers were significantly associated with disease progression, which is relevant given the increasing availability of these novel biomarkers in the clinical setting.

At baseline, the levels of CSF GFAP were increased in the hyposmic group, but these levels did not predict conversion to dementia. Previous research has indicated that CSF GFAP levels in PD are linked to cognitive decline, though findings about their relationship with dementia conversion has been inconsistent. One study found an association between GFAP and conversion to MCI, but no to dementia. In contrast, another study, which included a somewhat larger cohort and longer follow up, reported that higher CSF GFAP levels were associated with conversion to dementia [49,50]. It is possible that the present study was underpowered to find significant associations between CSF GFAP and dementia conversion given the small albeit significant difference in GFAP levels between the groups.

Previous studies have shown that CSF α-synuclein, is modestly decreased in PD [20,45], even at the earliest stages of disease [20]. However, the relationship between α-synuclein and cognitive decline has been inconsistent. Some studies have found that PD patients with higher levels of α-synuclein experience worse cognitive outcomes over time [45,51], while others have reported worse outcomes with lower levels [52]. One possible interpretation is that the decline in α-synuclein may begin already during the prodromal stages, and the association between increased α-synuclein with cognitive decline could indicate underlying synaptic dysfunction in these patients. It is possible that the lack of detected decline and association with dementia conversion in this study was due to insufficient statistical power.

Hyposmia is thought to indicate more severe extranigral disease [4]. Studies both in humans and mice have shown that the anterior olfactory nucleus, which receives projections from the olfactory bulb, projects to secondary brain structures like the entorhinal cortex. This complex network further includes both direct and indirect connections to other brain regions, such as the amygdala, substantia nigra, and brain stem nuclei, which are implicated in PD [53]. Longitudinal MRI studies in de novo PD patients have found that hyposmia is associated with a decrease in grey matter density and cortical volume over time in regions critical for cognitive function [54]. Additionally, hyposmia has been linked with reduced functional connectivity within the olfactory network and decreased functional connectivity in limbic regions of the brain, which may mediate the relationship between olfactory processing and cognitive function in PD [55].

Like other studies [4], hyposmia was a common feature present in 63% of the whole population. Smell identification worsened over the follow-up time in the whole cohort but was particularly pronounced in patients with PD with normal olfactory function at baseline. This aligns with previous findings [10], which indicate that olfactory dysfunction is not a fixed characteristic throughout the progression of the disease, showing the most pronounced decline in the early stages. In the current study, the observed decline in olfactory function was minor and most notable among participants who had a normal sense of smell at baseline. It is possible that the 12 item B-SIT used in this study lacked the sensitivity to detect subtle changes in the already hyposmic group. Additionally, as reported in earlier studies [9–16], no clear relationship was found between baseline olfactory function and various factors such as disease duration, cognitive symptoms, motor symptoms, autonomic symptoms and psychiatric symptoms.

CSF samples were analyzed using the Roche Elecsys® NeuroToolKit, a fully automated panel measuring neurodegeneration, glial activity, neuroinflammation and core AD markers, facilitating implementation and direct comparison between centers. While the panel has been previously studied in both patients with PD and AD [23–28], this is the first to investigate the CSF panel in relation to olfactory function. The findings were modest, revealing a small but significant increase in CSF GFAP in patients with PD and hyposmia compared to patients with PD and normal olfactory function (12.25 ± 3.87 vs 10.46 ± 3.68, p = 0.001). This result aligns with previous results from Li et al., who found a correlation between poorer performance on smell test and increased serum GFAP in PD, as well as an indirect effect on cognition through olfactory function [56]. CSF GFAP is considered a marker of astroglial activation and/or astrogliosis and has previously been shown to be elevated in several neurodegenerative disorders, including PD [57–59]. One possible interpretation of this finding is that increased levels of neuroinflammation in patients with PD and hyposmia. Further, α-synuclein is found not only in the neurons of the olfactory bulb but also in non-neuronal cells such as astroglia in the anterior olfactory nucleus, suggesting a potential link between GFAP to hyposmia [60]. However, it is more likely that the elevated GFAP levels reflect a more aggressive disease course in hyposmic PD patients, leading to a stronger inflammatory response. Interestingly, no significant differences in other CSF markers, including core AD markers, were observed between the groups, suggesting that olfactory dysfunction is not necessarily linked to concomitant AD-pathology.

The main strength of this study lies in its well-characterized prospective cohort of PD patients who were followed for up to 11 years. The patients were predominantly recruited from clinical settings, which suggests that the findings may be generalizable. However, similar to other studies in olfaction and PD [9], sample sizes within each diagnostic group were limited, and a validation cohort was not included, which restricts the interpretation of the results. Employing a more extensive range of cognitive assessments could have improved differentiation between cognitive domains related to olfactory dysfunction and may have enhanced sensitivity to cognitive decline over time. Additionally, the 12-item B-SIT may not be sensitive enough to detect subtle changes in the already hyposmic group, indicating that a more comprehensive smelling test could have strengthened the study. While the CSF biomarker panel is validated and reliable, confirming amyloid status through amyloid PET imaging or post-mortem examination would have been a possible improvement. Further, using the recently developed plasma biomarkers would have been more novel than CSF biomarkers, even though the latter are still dominating clinical practice.

## Conclusions

Olfactory dysfunction and amyloid-positivity are independently associated with an increased risk of both cognitive and motor function decline, ultimately increasing the likelihood of progression to dementia over time. Notably, other clinical variables, including autonomic or psychiatric symptoms, do not appear to offer additional value in predicting disease progression. Similarly, novel CSF markers also lack additional predictive value. These findings underscore the importance of olfactory assessments and amyloid status in predicting clinical outcomes in patients with PD. These insights could be implemented into clinical practice; however further research involving diverse and larger cohorts is essential to validate these associations and enhance our understanding of disease progression.

## Supporting information

Fig S1 CSF biomarkers at baseline in participants with Parkinson’s disease and normal olfactory function and hyposmia. (TIFF)

Fig S2 Longitudinal trajectories of CSF markers in participants with Parkinson’s disease with normal olfactory function and hyposmia. Bold lines represent the predicted trajectory in each group. CSF markers were log-transformed and scaled.
(TIFF)

Table S1 Cox proportional hazard analyses. Each variable has been analyzed only corrected for age and gender.
(DOCX)

## References

[1] Poewe W, Seppi K, Tanner CM, Halliday GM, Brundin P, Volkmann J, et al. Parkinson disease. Nat Rev Dis Primers. 2017;3:17013. doi: 10.1038/nrdp.2017.13 28332488

[2] Ou Z, Pan J, Tang S, Duan D, Yu D, Nong H, et al. Global trends in the incidence, prevalence, and years lived with disability of Parkinson’s disease in 204 countries/territories from 1990 to 2019. Front Public Health. 2021;9:776847. doi: 10.3389/fpubh.2021.776847 34950630 PMC8688697

[3] Postuma RB, Berg D, Stern M, Poewe W, Olanow CW, Oertel W, et al. MDS clinical diagnostic criteria for Parkinson’s disease. Mov Disord. 2015;30(12):1591–601. doi: 10.1002/mds.26424 26474316

[4] Fullard ME, Morley JF, Duda JE. Olfactory dysfunction as an early biomarker in parkinson’s disease. Neurosci Bull. 2017;33(5):515–25. doi: 10.1007/s12264-017-0170-x 28831680 PMC5636737

[5] Masala C, Solla P, Liscia A, Defazio G, Saba L, Cannas A, et al. Correlation among olfactory function, motors’ symptoms, cognitive impairment, apathy, and fatigue in patients with Parkinson’s disease. J Neurol. 2018;265(8):1764–71. doi: 10.1007/s00415-018-8913-9 29804147

[6] Del Tredici K, Rüb U, De Vos RAI, Bohl JRE, Braak H. Where does parkinson disease pathology begin in the brain?. J Neuropathol Exp Neurol. 2002;61(5):413–26. doi: 10.1093/jnen/61.5.413 12030260

[7] Braak H, Del Tredici K, Rüb U, de Vos RAI, Jansen Steur ENH, Braak E. Staging of brain pathology related to sporadic Parkinson’s disease. Neurobiol Aging. 2003;24(2):197–211. doi: 10.1016/s0197-4580(02)00065-9 12498954

[8] Beach TG, Adler CH, Zhang N, Serrano GE, Sue LI, Driver-Dunckley E, et al. Severe hyposmia distinguishes neuropathologically confirmed dementia with Lewy bodies from Alzheimer’s disease dementia. PLoS One. 2020;15(4):e0231720. doi: 10.1371/journal.pone.0231720 32320406 PMC7176090

[9] Morley JF, Duda JE. Neuropsychological correlates of olfactory dysfunction in Parkinson’s disease. J Neurol Sci. 2011;310(1–2):228–30. doi: 10.1016/j.jns.2011.05.030 21658728

[10] Ercoli T, Masala C, Cadeddu G, Mascia MM, Orofino G, Gigante AF, et al. Does olfactory dysfunction correlate with disease progression in Parkinson’s disease? A systematic review of the current literature. Brain Sci. 2022;12(5):513. doi: 10.3390/brainsci12050513 35624900 PMC9139278

[11] He R, Zhao Y, He Y, Zhou Y, Yang J, Zhou X, et al. Olfactory dysfunction predicts disease progression in Parkinson’s disease: a longitudinal study. Front Neurosci. 2020;14:569777. doi: 10.3389/fnins.2020.569777 33381006 PMC7768001

[12] Berendse HW, Roos DS, Raijmakers P, Doty RL. Motor and non-motor correlates of olfactory dysfunction in Parkinson’s disease. J Neurol Sci. 2011;310(1–2):21–4. doi: 10.1016/j.jns.2011.06.020 21705022

[13] Bohnen NI, Müller MLTM, Kotagal V, Koeppe RA, Kilbourn MA, Albin RL, et al. Olfactory dysfunction, central cholinergic integrity and cognitive impairment in Parkinson’s disease. Brain. 2010;133(Pt 6):1747–54. doi: 10.1093/brain/awq079 20413575 PMC2877903

[14] Damholdt MF, Borghammer P, Larsen L, Ostergaard K. Odor identification deficits identify Parkinson’s disease patients with poor cognitive performance. Mov Disord. 2011;26(11):2045–50. doi: 10.1002/mds.23782 21638326

[15] Fullard ME, Tran B, Xie SX, Toledo JB, Scordia C, Linder C, et al. Olfactory impairment predicts cognitive decline in early Parkinson’s disease. Parkinsonism Relat D. 2016;25:45–51. doi: 10.1016/j.parkreldis.2016.02.013 26923521 PMC4825674

[16] Barone P, Antonini A, Colosimo C, Marconi R, Morgante L, Avarello TP, et al. The PRIAMO study: A multicenter assessment of nonmotor symptoms and their impact on quality of life in Parkinson’s disease. Mov Disord. 2009;24(11):1641–9. doi: 10.1002/mds.22643 19514014

[17] Domellöf ME, Lundin K-F, Edström M, Forsgren L. Olfactory dysfunction and dementia in newly diagnosed patients with Parkinson’s disease. Parkinsonism Relat D. 2017;38:41–7. doi: 10.1016/j.parkreldis.2017.02.017 28242255

[18] Baba T, Kikuchi A, Hirayama K, Nishio Y, Hosokai Y, Kanno S, et al. Severe olfactory dysfunction is a prodromal symptom of dementia associated with Parkinson’s disease: a 3 year longitudinal study. Brain. 2012;135(Pt 1):161–9. doi: 10.1093/brain/awr321 22287381

[19] Guo P, Wang R-D, Lian T-H, Ding D-Y, Zhang Y-N, Zhang W-J, et al. Olfactory dysfunction and its association with neuropathologic proteins in cerebrospinal fluid from patients with parkinson disease. Front Aging Neurosci. 2020;12:594324. doi: 10.3389/fnagi.2020.594324 33362530 PMC7759606

[20] Mollenhauer B, Caspell-Garcia CJ, Coffey CS, Taylor P, Singleton A, Shaw LM, et al. Longitudinal analyses of cerebrospinal fluid α-Synuclein in prodromal and early Parkinson’s disease. Mov Disord. 2019;34(9):1354–64. doi: 10.1002/mds.27806 31361367 PMC7098385

[21] MacMahon Copas AN, McComish SF, Fletcher JM, Caldwell MA. The pathogenesis of Parkinson’s disease: a complex interplay between astrocytes, microglia, and T lymphocytes?. Front Neurol. 2021;12:666737. doi: 10.3389/fneur.2021.666737 34122308 PMC8189423

[22] Guo Y, Wei X, Yan H, Qin Y, Yan S, Liu J, et al. TREM2 deficiency aggravates α-synuclein-induced neurodegeneration and neuroinflammation in Parkinson’s disease models. FASEB J. 2019;33(11):12164–74. doi: 10.1096/fj.201900992R 31370707 PMC6902667

[23] Milà-Alomà M, Salvadó G, Gispert JD, Vilor-Tejedor N, Grau-Rivera O, Sala-Vila A, et al. Amyloid beta, tau, synaptic, neurodegeneration, and glial biomarkers in the preclinical stage of the Alzheimer’s continuum. Alzheimers Dement. 2020;16(10):1358–71. doi: 10.1002/alz.12131 32573951 PMC7586814

[24] Van Hulle C, Jonaitis EM, Betthauser TJ, Batrla R, Wild N, Kollmorgen G, et al. An examination of a novel multipanel of CSF biomarkers in the Alzheimer’s disease clinical and pathological continuum. Alzheimers Dement. 2021;17(3):431–45. doi: 10.1002/alz.12204 33336877 PMC8016695

[25] Bos I, Vos S, Verhey F, Scheltens P, Teunissen C, Engelborghs S, et al. Cerebrospinal fluid biomarkers of neurodegeneration, synaptic integrity, and astroglial activation across the clinical Alzheimer’s disease spectrum. Alzheimers Dement. 2019;15(5):644–54. doi: 10.1016/j.jalz.2019.01.004 30853464

[26] Salvadó G, Milà-Alomà M, Shekari M, Minguillon C, Fauria K, Niñerola-Baizán A, et al. Cerebral amyloid-β load is associated with neurodegeneration and gliosis: mediation by p-tau and interactions with risk factors early in the Alzheimer’s continuum. Alzheimers Dement. 2021;17(5):788–800. doi: 10.1002/alz.12245 33663013 PMC8252618

[27] Bartl M, Dakna M, Galasko D, Hutten SJ, Foroud T, Quan M, et al. Biomarkers of neurodegeneration and glial activation validated in Alzheimer’s disease assessed in longitudinal cerebrospinal fluid samples of Parkinson’s disease. PLoS One. 2021;16(10):e0257372. doi: 10.1371/journal.pone.0257372 34618817 PMC8496858

[28] Qin Q, Wan H, Wang D, Li J, Qu Y, Zhao J, et al. The Association of CSF sTREM2 with cognitive decline and its dynamic change in Parkinson’s disease: analysis of the ppmi cohort. Front Aging Neurosci. 2022;14:892493. doi: 10.3389/fnagi.2022.892493 35783125 PMC9245456

[29] Gelb DJ, Oliver E, Gilman S. Diagnostic criteria for Parkinson disease. Arch Neurol. 1999;56(1):33–9. doi: 10.1001/archneur.56.1.33 9923759

[30] Doty RL, Marcus A, Lee WW. Development of the 12-item cross-cultural smell identification test (CC-SIT). Laryngoscope. 1996;106(3 Pt 1):353–6. doi: 10.1097/00005537-199603000-00021 8614203

[31] Doty RL. The Smell Identification Test Administration Manual. Haddon Heights, NJ: Sensonics, Inc. 1995.

[32] Folstein MF, Folstein SE, McHugh PR. “Mini-mental state”. A practical method for grading the cognitive state of patients for the clinician. J Psychiatr Res. 1975;12(3):189–98. doi: 10.1016/0022-3956(75)90026-6 1202204

[33] Mohs RC, Knopman D, Petersen RC, Ferris SH, Ernesto C, Grundman M, et al. Development of cognitive instruments for use in clinical trials of antidementia drugs: additions to the Alzheimer’s Disease Assessment Scale that broaden its scope. The Alzheimer’s Disease Cooperative Study. Alzheimer Dis Assoc Disord. 1997;11 Suppl 2:S13-21. doi: 10.1097/00002093-199700112-00003 9236948

[34] Kvitting AS, Wimo A, Johansson MM, Marcusson J. A quick test of cognitive speed (AQT): usefulness in dementia evaluations in primary care. Scand J Prim Health Care. 2013;31(1):13–9. doi: 10.3109/02813432.2012.751699 23293859 PMC3587304

[35] Fahn S, Elton R, Committee UD. Unified Parkinson’s disease rating scale. Florham Park, NJ: Macmillan Healthcare Information. 1987.

[36] Goetz CG, Poewe W, Rascol O, Sampaio C, Stebbins GT, Counsell C, et al. Movement disorder society task force report on the hoehn and yahr staging scale: status and recommendations. Mov Disord. 2004;19(9):1020–8. doi: 10.1002/mds.20213 15372591

[37] Visser M, Marinus J, Stiggelbout AM, Van Hilten JJ. Assessment of autonomic dysfunction in Parkinson’s disease: the SCOPA-AUT. Mov Disord. 2004;19(11):1306–12. doi: 10.1002/mds.20153 15390007

[38] Jankovic J, McDermott M, Carter J, Gauthier S, Goetz C, Golbe L, et al. Variable expression of Parkinson’s disease: a base-line analysis of the DATATOP cohort. The Parkinson Study Group. Neurology. 1990;40(10):1529–34. doi: 10.1212/wnl.40.10.1529 2215943

[39] Zigmond AS, Snaith RP. The hospital anxiety and depression scale. Acta Psychiatr Scand. 1983;67(6):361–70. doi: 10.1111/j.1600-0447.1983.tb09716.x 6880820

[40] Palmqvist S, Zetterberg H, Blennow K, Vestberg S, Andreasson U, Brooks DJ, et al. Accuracy of brain amyloid detection in clinical practice using cerebrospinal fluid β-amyloid 42: a cross-validation study against amyloid positron emission tomography. JAMA Neurol. 2014;71(10):1282–9. doi: 10.1001/jamaneurol.2014.1358 25155658

[41] Quadalti C, Palmqvist S, Hall S, Rossi M, Mammana A, Janelidze S, et al. Clinical effects of Lewy body pathology in cognitively impaired individuals. Nat Med. 2023;29(8):1964–70. doi: 10.1038/s41591-023-02449-7 37464058 PMC10427416

[42] Mertens AT, Santo JB, Markopoulou K, Chase BA. Cognitive processes that indirectly affect olfactory dysfunction in Parkinson’s disease. Clin Park Relat Disord. 2019;1:13–20. doi: 10.1016/j.prdoa.2019.07.003 34316593 PMC8288748

[43] Markopoulou K, Chase BA, Robowski P, Strongosky A, Narożańska E, Sitek EJ, et al. Assessment of olfactory function in mapt-associated neurodegenerative disease reveals odor-identification irreproducibility as a non-disease-specific, general characteristic of olfactory dysfunction. PLoS One. 2016;11(11):e0165112. doi: 10.1371/journal.pone.0165112 27855167 PMC5113898

[44] Siderowf A, Xie SX, Hurtig H, Weintraub D, Duda J, Chen-Plotkin A, et al. CSF amyloid {beta} 1-42 predicts cognitive decline in Parkinson disease. Neurology. 2010;75(12):1055–61. doi: 10.1212/WNL.0b013e3181f39a78 20720189 PMC2942062

[45] Hall S, Surova Y, Öhrfelt A, Zetterberg H, Lindqvist D, Hansson O. CSF biomarkers and clinical progression of Parkinson disease. Neurology. 2015;84(1):57–63. doi: 10.1212/WNL.0000000000001098 25411441 PMC4336091

[46] Irwin DJ, Fedler J, Coffey CS, Caspell-Garcia C, Kang JH, Simuni T, et al. Evolution of Alzheimer’s disease cerebrospinal fluid biomarkers in early Parkinson’s disease. Ann Neurol. 2020;88(3):574–87. doi: 10.1002/ana.25811 32542885 PMC7497251

[47] Gomperts SN, Locascio JJ, Rentz D, Santarlasci A, Marquie M, Johnson KA, et al. Amyloid is linked to cognitive decline in patients with Parkinson disease without dementia. Neurology. 2013;80(1):85–91. doi: 10.1212/WNL.0b013e31827b1a07 23243071 PMC3589197

[48] Mihaescu AS, Valli M, Uribe C, Diez-Cirarda M, Masellis M, Graff-Guerrero A, et al. Beta amyloid deposition and cognitive decline in Parkinson’s disease: a study of the PPMI cohort. Mol Brain. 2022;15(1):79. doi: 10.1186/s13041-022-00964-1 36100909 PMC9472347

[49] Liu Y, Wang J, Ning F, Wang G, Xie A. Longitudinal correlation of cerebrospinal fluid GFAP and the progression of cognition decline in different clinical subtypes of Parkinson’s disease. Clin Transl Sci. 2024;17(12):e70111. doi: 10.1111/cts.70111 39676304 PMC11647050

[50] Liu T, Zuo H, Ma D, Song D, Zhao Y, Cheng O. Cerebrospinal fluid GFAP is a predictive biomarker for conversion to dementia and Alzheimer’s disease-associated biomarkers alterations among de novo Parkinson’s disease patients: a prospective cohort study. J Neuroinflammation. 2023;20(1):167. doi: 10.1186/s12974-023-02843-5 37475029 PMC10357612

[51] Stewart T, Liu C, Ginghina C, Cain KC, Auinger P, Cholerton B, et al. Cerebrospinal fluid α-synuclein predicts cognitive decline in Parkinson disease progression in the DATATOP cohort. Am J Pathol. 2014;184(4):966–75. doi: 10.1016/j.ajpath.2013.12.007 24625392 PMC3969999

[52] Skogseth RE, Bronnick K, Pereira JB, Mollenhauer B, Weintraub D, Fladby T, et al. Associations between cerebrospinal fluid biomarkers and cognition in early untreated Parkinson’s disease. J Parkinsons Dis. 2015;5(4):783–92. doi: 10.3233/JPD-150682 26599300 PMC5031486

[53] Ubeda-Bañon I, Saiz-Sanchez D, de la Rosa-Prieto C, Martinez-Marcos A. α-Synuclein in the olfactory system in Parkinson’s disease: role of neural connections on spreading pathology. Brain Struct Funct. 2014;219(5):1513–26. doi: 10.1007/s00429-013-0651-2 24135772

[54] Kawabata K, Bagarinao E, Seppi K, Poewe W. Longitudinal brain changes in Parkinson’s disease with severe olfactory deficit. Parkinsonism Relat Disord. 2024;122:106072. doi: 10.1016/j.parkreldis.2024.106072 38430690

[55] Xing Y, Zhou H, Chen S, Wang Y, Ren J, Cao Y, et al. Olfactory network disruptions as mediators of cognitive impairment in De Novo Parkinson’s disease. CNS Neurosci Ther. 2025;31(1):e70198. doi: 10.1111/cns.70198 39803685 PMC11726119

[56] Li F-J, Li Y-D-Y, Liu X, Zu J, Zhang W, Xiao Q-H, et al. Serum biomarkers of olfactory identification deficits in patients with Parkinson’s disease. Acta Neurologica Scandinavica. 2023;2023:1–12. doi: 10.1155/2023/8860125

[57] Schulz I, Kruse N, Gera RG, Kremer T, Cedarbaum J, Barbour R, et al. Systematic assessment of 10 biomarker candidates focusing on α-synuclein-related disorders. Mov Disord. 2021;36(12):2874–87. doi: 10.1002/mds.28738 34363416

[58] Süssmuth SD, Uttner I, Landwehrmeyer B, Pinkhardt EH, Brettschneider J, Petzold A, et al. Differential pattern of brain-specific CSF proteins tau and amyloid-β in Parkinsonian syndromes. Mov Disord. 2010;25(9):1284–8. doi: 10.1002/mds.22895 20589870

[59] Bellaver B, Ferrari-Souza JP, Uglione da Ros L, Carter SF, Rodriguez-Vieitez E, Nordberg A, et al. Astrocyte biomarkers in Alzheimer disease: a systematic review and meta-analysis. Neurology. 2021;96(24):e2944–55. doi: 10.1212/WNL.0000000000012109 33952650

[60] Stevenson TJ, Murray HC, Turner C, Faull RLM, Dieriks BV, Curtis MA. α-synuclein inclusions are abundant in non-neuronal cells in the anterior olfactory nucleus of the Parkinson’s disease olfactory bulb. Sci Rep. 2020;10(1):6682. doi: 10.1038/s41598-020-63412-x 32317654 PMC7174302

