# Supplementary figures and images for: Olfactory dysfunction and amyloid-positivity in Parkinson’s disease—longitudinal analysis of cognitive decline and cerebrospinal fluid markers
Source: PLoS One. 2025 Aug 21;20(8):e0325560. doi: 10.1371/journal.pone.0325560 (PMC12370119; doi:10.1371/journal.pone.0325560)

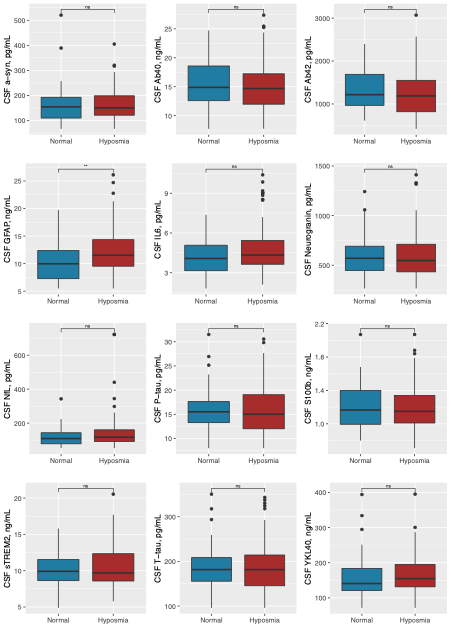

Supplement: Fig S1 — (TIFF) [file pone.0325560.s001.tiff]

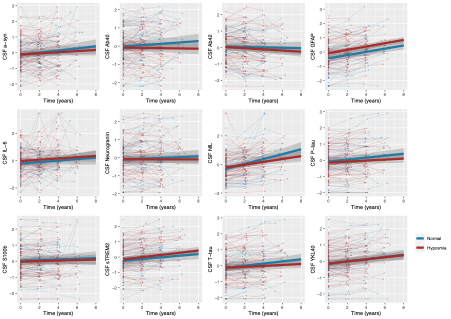

Supplement: Fig S2 — Bold lines represent the predicted trajectory in each group. CSF markers were log-transformed and scaled. (TIFF) [file pone.0325560.s002.tiff]
