# Supplementary material for: Olfactory dysfunction and amyloid-positivity in Parkinson’s disease—longitudinal analysis of cognitive decline and cerebrospinal fluid markers
Source: PLoS One. 2025 Aug 21;20(8):e0325560. doi: 10.1371/journal.pone.0325560 (PMC12370119; doi:10.1371/journal.pone.0325560)
Supplement: Table S1 — Each variable has been analyzed only corrected for age and gender. (DOCX) [file pone.0325560.s003.docx]

**Table S1.** **Cox proportional hazard analyses.** Each variable has been analyzed only corrected for age and gender.

| **Variable** | **Beta** | **SE** | **HR (95% CI)** | **p-value** |
| --- | --- | --- | --- | --- |
| Age, baseline | 0.08 | 0.02 | 1.09 (1.05-1.13) | <0.0001 |
| Amyloid positive*, yes | 1.37 | 0.33 | 3.94 (2.06-7.52) | <0.0001 |
| CSF Aβ42/ Aβ40 | -0.46 | 0.14 | 0.63 (0.48-0.83) | <0.001 |
| B-SIT, score | -0.19 | 0.06 | 0.83 (0.74-0.93) | 0.002 |
| SCOPA-AUT, score | 0.05 | 0.02 | 1.06 (1.02-1.10) | 0.004 |
| AQT colour form | 0.02 | 0.01 | 1.02 (1.00-1.04) | 0.004 |
| HADS anxiety, subscore | 0.09 | 0.04 | 1.09 (1.02-1.17) | ns |
| ADAS-Cog ten-word delayed recall, score | 0.17 | 0.07 | 1.19 (1.04-1.36) | ns |
| CSF Aβ42, pg/mL | -0.38 | 0.15 | 0.69 (0.51-0.93) | ns |
| APOE-e4, carrier, yes | 0.69 | 0.29 | 2.0 (1.12-3.52) | ns |
| CSF NfL pg/mL | 0.41 | 0.20 | 1.51 (1.01-2.25) | ns |
| MMSE, score | -0.12 | 0.07 | 0.89 (0.77-1.02) | ns |
| Orthostatic hypotension, yes | 0.50 | 0.30 | 1.64 (0.91-2.97) | ns |
| HADS depression, subscore | 0.06 | 0.04 | 1.07 (0.99-1.15) | ns |
| Smoker, yes | 1.13 | 0.76 | 3.09 (0.70-13.60) | ns |
| Hoehn & Yahr, score | 0.25 | 0.18 | 1.29 (0.90-1.83) | ns |
| CSF T-tau, pg/mL | 0.24 | 0.17 | 1.27 (0.91-1.77) | ns |
| Disease duration, years | 0.03 | 0.03 | 1.03 (0.98-1.09) | ns |
| CSF Neurogranin, pg/mL | 0.19 | 0.16 | 1.21 (0.88-1.66) | ns |
| CSF P-tau, pg/mL | 0.16 | 0.17 | 1.17 (0.84-1.64) | ns |
| CSF α-synuclein, pg/mL | 0.14 | 0.15 | 1.15 (0.86-1.56) | ns |
| CSF S100b, ng/mL | -0.12 | 0.16 | 0.89 (0.65-1.21) | ns |
| CSF IL-6, pg/mL | -0.09 | 0.16 | 0.92 (0.67-1.26) | ns |
| CSF GFAP, ng/mL | 0.10 | 0.19 | 1.11 (0.76-1.61) | ns |
| Sex, female | -0.16 | 0.30 | 0.86 (0.48-1.53) | ns |
| UPDRS-III, score | 0.01 | 0.01 | 1.01 (0.98-1.03) | ns |
| CSF sTREM2, ng/mL | 0.06 | 0.17 | 1.06 (0.76-1.49) | ns |
| CSF YKL-40, ng/mL | -0.06 | 0.17 | 0.95 (0.67-1.33) | ns |
| Education, years | -0.01 | 0.04 | 0.99 (0.93-1.06) | ns |
| CSF Aβ40, ng/mL | -0.01 | 0.15 | 0.99 (0.73-1.33) | ns |
